# Characterization of a Spontaneous Novel Mutation in the *NPC2* Gene in a Cat Affected by Niemann Pick Type C Disease

**DOI:** 10.1371/journal.pone.0112503

**Published:** 2014-11-14

**Authors:** Stefania Zampieri, Ezio Bianchi, Carlo Cantile, Roberta Saleri, Bruno Bembi, Andrea Dardis

**Affiliations:** 1 Regional Coordinator Centre for Rare Diseases, University Hospital Santa Maria della Misericordia, Udine, Italy; 2 Department of Veterinary Medical Sciences, University of Parma, Parma, Italy; 3 Department of Veterinary Sciences, University of Pisa, Pisa, Italy; The University of New South Wales, Australia

## Abstract

Niemann-Pick C disease (NPC) is an autosomal recessive lysosomal storage disorder characterized by accumulation of unesterified cholesterol and other lipids within the lysosomes due to mutation in *NPC1* or *NPC2* genes. A feline model of NPC carrying a mutation in *NPC1* gene has been previously described. We have identified two kittens affected by NPC disease due to a mutation in *NPC2* gene. They manifested with tremors at the age of 3 months, which progressed to dystonia and severe ataxia. At 6 months of age cat 2 was unable to stand without assistance and had bilaterally reduced menace response. It died at the age of 10 months. Post-mortem histological analysis of the brain showed the presence of neurons with cytoplasmic swelling and vacuoles, gliosis of the substantia nigra and degeneration of the white matter. Spheroids with accumulation of ubiquitinated aggregates were prominent in the cerebellar cortex. Purkinje cells were markedly reduced in number and they showed prominent intracytoplasmic storage. Scattered perivascular aggregates of lymphocytes and microglial cells proliferation were present in the thalamus and midbrain. Proliferation of Bergmann glia was also observed. In the liver, hepatocytes were swollen because of accumulation of small vacuoles and foamy Kupffer cells were also detected. Foamy macrophages were observed within the pulmonary interstitium and alveoli as well. At 9 months cat 1 was unable to walk, developed seizures and it was euthanized at 21 months. Filipin staining of cultured fibroblasts showed massive storage of unesterified cholesterol. Molecular analysis of *NPC1* and *NPC2* genes showed the presence of a homozygous intronic mutation (c.82+5G>A) in the *NPC2* gene. The subsequent analysis of the mRNA showed that the mutation causes the retention of 105 bp in the mature mRNA, which leads to the in frame insertion of 35 amino acids between residues 28 and 29 of NPC2 protein (p.G28_S29ins35).

## Introduction

Niemann Pick C [NPC-MIM 257220; MIM607625] disease is a neurodegenerative lysosomal storage disorder due to mutations in *NPC1* or *NPC2* genes. Both NPC1 and NPC2 proteins are involved in the intracellular trafficking of cholesterol and other lipids. Thus, the deficiency of either of them leads to the accumulation of the endocytosed unesterified cholesterol, gangliosides and other lipids within the lysosome/late endosome compartment [1].

Approximately 95% of NPC patients present mutations in *NPC1* gene (MIM 607623; chr 18q11–q12) [2], [3], which encodes a membrane glycoprotein of 1,278 amino acids containing 13 transmembrane domains and localized in late endosomes. The other 5% of patients present mutations in *NPC2* gene (MIM 601015; chr 14q24.3) [4] encoding a soluble 151 amino acid protein that is present in the lumen of lysosomes.

In humans, NPC disease presents a highly variable phenotype ranging from fetal to adult age. Apart from patients affected by the perinatal form of the disease, which manifests at birth or within the first month of life with severe visceral involvement (fetal hydrops, ascites, neonatal cholestasis, liver failure and/or specific pulmonary disease), often leading to death [5], [6], most patients develop a progressive and fatal neurological disease. In these patients, even if initial manifestations may be systemic, with liver and spleen enlargement, neurological, or psychiatric, the disease has been classified according to the age at onset of neurological symptoms in a severe infantile form (onset before 2 y of age), a late infantile form (onset between 3–5 y of age), a juvenile form (onset between 5 and 16 y) and an adult form (onset at age>16 y) [1], [7].

Patients with mutations in *NPC1* or *NPC2* are almost identical from the clinical point of view. However a high incidence of severe respiratory insufficiency has been reported in infants having mutations in the *NPC2* gene [8]. Furthermore, in two patients' lung lavage, radiology and histology showed signs of pulmonary alveolar lipoproteinosis [9], [10].

Two mouse models of NPC1 and one of NPC2 disease have been described. Both mouse models of NPC1 recapitulate the main features of human pathology. However, while the naturally occurring NPC1 mouse (BALB\c NPC) presents a very severe phenotype [11], the Npc1 (nmf164) mouse, which carries the c.3163A>G mutation, displays a slower development of the NPC phenotype [12].

The phenotype of the transgenic NPC2 hypomorph mouse, and the BALB\c NPC are very similar albeit with slightly later onset and slower progression [13].

In addition, a canine and a feline NPC1 models, both spontaneous, are known [14], [15]. In particular, the feline model, which presents a missense mutation c.2864G>C (C955S) [15], has been exhaustively characterized and is phenotypically, morphologically, and biochemically similar to human juvenile neurological form of NPC [16].

In this study we describe two kittens (one male and one female) from the same litter affected by NPC disease caused by a mutation in the *NPC2* gene.

## Materials and Methods

All interventions were performed as part of routine monitoring and care. Skin biopsy and post-mortem tissues of affected kittens were obtained in the course of diagnostic work-up while skin tissue from a healthy cat was obtained during surgical sterilization.

The cat euthanized (cat1) was pre anesthetized with dexmedetomidine (40 ug/kg), then a deep plane of general anesthesia was induced with propofol. An intravenous infusion of a solution of Embutramide, Mebezonium iodide and Tetracaine hydrochloride (Tanax - Intervet Italia) was performed following the doses indicated by the manufacturer.

The local ethics committee was notified and because it controls animal experimentation and not non-experimental clinical veterinary practices (Dlgs N.26 4/3/2014), the committee waived approval.

All the procedures were performed in agreement with FVE code of good veterinary practice (http://www.fve.org/news/publications/pdf/gvp.pdf). In all cases written consent was obtained from animal's owners.

### Cell culture and filipin staining

Fibroblasts from an affected cat and a normal control were obtained from skin biopsies. Briefly, skin tissues were washed three times in PBS containing antibiotics and then three times with Dulbecco's modified Eagle's medium (DMEM; Invitrogen, Grand Island, NY, USA) supplemented with 10% foetal bovine serum (FBS; Invitrogen) and antibiotics.

After washing, the samples were placed in 60-mm culture dishes containing DMEM supplemented with 10% FBS and antibiotics and cultured at 37°C in a humidified atmosphere containing 5% CO_2_. The culture medium was changed every 2 days. When cells reached 90% confluence were trypsinized and seeded into 6 well plates or on coverslips, or frozen at −80°C using a Mr Frosty (Sigma) device gradient and stored in liquid nitrogen.

Filipin staining was performed using the method described by Blanchette-Mackie et al [17].

### Mutational analysis

Genomic DNA and total RNA were extracted from cultured fibroblasts using QIAamp DNA blood Mini Kit (Qiagen GmbH, Hilden, Germany) and Rneasy mini Kit (Qiagen GmbH, Hilden, Germany), respectively.

The exonic and the flanking intronic sequences of the feline *NPC1* and *NPC2* genes (NC_018734.1 and NC_018728.1, respectively) were PCR amplified using specific primers. PCR primers designed using the Primer3 program, and PCR conditions are available upon request.

For RT-PCR analysis of the *NPC2* mRNA the first strand cDNA was synthesized using random hexamer primers and subsequent amplified using primers located in the 5′UTR and 3′UTR region.

PCR and RT-PCR products were analyzed by automated sequencing (ABI Prism 3500xl genetic analyzer). Putative mutations were confirmed by sequencing duplicate PCR products and by the DNA analysis from the mother.

### Western bot

Twenty micrograms of protein extracts were resolved on 15% SDS PAGE gels and transferred to nitrocellulose membranes (Schleicher and Schuell, Keene, NH, USA). After overnight blocking with 5% nonfat dry milk in PBS-Tween 0.1% (PBS-T), the membranes were probed with anti-NPC2 polyclonal antibody (Sigma, St Louis, MO, USA) overnight at 4°C. Anti-rabbit HPR conjugated antibody was used as a secondary antibody. Immunoreactive bands were detected by enhanced chemiluminescence ECL (Amersham). The signals were normalized to those obtained for actin using a polyclonal anti-actin antibody (Sigma, St Louis, MO, USA).

### Structural 3D analysis

A multiple sequence alignment of NPC2 homologs was made using Clustal-Omega [18]. The amino acid sequences of wild type and mutant NPC2 protein were used to predict the interactions due to disulfide bonds through the DiANNA website (http://clavius.bc.edu/~clotelab/DiANNA/). The image of the bovine NPC2 (bNPC2) crystal structure, as well as the identification of amino acid residues, was done using the SWISS-PDB VIEWER (http://www.expasy.org/spdbv/; [19]).

### Histology

The brain and spinal cord and samples of major organs were fixed in phosphate buffered 4% formalin solution. Tissue samples were routinely processed for histology and sections were stained with haematoxylin and eosin (HE), periodic acid-Schiff (PAS), Luxol fast blue (LFB), crystal violet for Nissl substance, and Bielschowsky silver stain. Selected brain sections were stained with the peroxidase-anti-peroxidase (PAP) method using a polyclonal antibody against glial fibrillary acidic protein (GFAP; 1∶1000, Dako, Carpinteria, USA), CD3 (1∶100, Dako, Carpinteria, USA), ubiquitin (1∶1000, Dako, Carpinteria, USA) and calbindin (1∶299, Santa Cruz Biotechnology, USA).

## Results

### Clinical findings

Two kittens (1 male and 1 female) from a litter of 5 were evaluated for progressive neurologic signs. In both cats clinical signs had an insidious onset at around 3 month of age, characterized by intention tremors and truncal ataxia. Neurological manifestations, suggestive of a cerebellar involvement, had a slow progression and led to dystonia and worsening of gait over time. At 6 months of age the female (cat 1) had a prayer-like posture and was still ambulatory with hypermetria of the hind limbs, while the male (cat 2) wasn't able to stand without assistance and had bilaterally reduced menace response. Cat 1 underwent a complete diagnostic work-up and was clinically monitored during the course of the disease. At 5 months of age an abdominal ultrasonography was unremarkable, a complete blood cell count, serum biochemistry panel (Table 1) and urine analysis showed only a mild increase in ALT (51 U/l; normal values 5–45 U/l) and CK (123 U/l; normal values 10–100 U/l). A titre of 1∶1024 for anti-Toxoplasma gondii IgG was also found. The ALT values at 7 and 19 months were 102 and 42 U/l, respectively. At 6 months of age the results of a magnetic resonance (MR) of the brain were unremarkable, while the brainstem auditory evoked potentials (BAEPs) showed a prolonged I–V interpeak latency.

**Table 1:** Laboratory parameters.

	5 months	7 months	19 months	Reference values
RBC 10∧6 µl	8.19	9.17	8.61	5.0–10.0
WBC 10∧3 µl	14.3	11.4	16.4	6.0–17.0
Hgb g/dl	11.8	12.7	13.0	9–15
Hct %	31.3	34.5	38.4	30–45
MCV fl	38.2	37.6	44.6	40–54
RDW %	22.4	21.8	22.0	14–18
MCHC g/dl	37.7	36.7	34.0	31–36
MCH pg	14.4	13.8	15.1	14–18
PLT 10∧3 µl	448	554	260	250–750
Total Bilirubin mg/dl	0.11	0.01	0.05	0.01–0.2
Fasting Bile acids µmol/L			3.99	<10
ALT U/l	51	102	42	5–45
GGT U/l	0.1	0.2	0.1	0.1–5
Glucose mg/dl	81	70	93	60–130
BUN mg/dl	48.5	45	37.3	20–65
Creatinine mg/dl	0.53	0.65	0.45	0.1–1.6
ALP U/l	73	48	19	10–100
Total Protein g/dl	6.23	6.33	6.95	6.5–8.5
Albumin g/dl	3.11	3.26	3.31	2.3–3.3
Total Cholesterol mg/dl		81	120	75–150
Triglycerides mg/dl		33	72	50–100
Total Ca mg/dl	9.98			8–12
Ph mg/dl	7.36			3–5
Na mEq/l	149.9			145–158
Cl mEq/l	115.7			110–130
K mEq/l	3.78			3–4.8
CK U/l	123			10–100
LDH U/l	50			10–150

Cat 2 died at the age of 10 months for respiratory paralysis. The brain and spinal cord and samples of major organs were collected. At 9 months cat 1 was unable to walk and developed seizures. It was humanely euthanized a year later upon request of the owner after developing severe dysphagia for the poor prognosis. Neither of the cats showed clinical signs of pulmonary disease over the course of their life, and serial clinical evaluations of respiratory function revealed no obvious abnormalities

### Biochemical and genetic analysis

**Figure 1:**
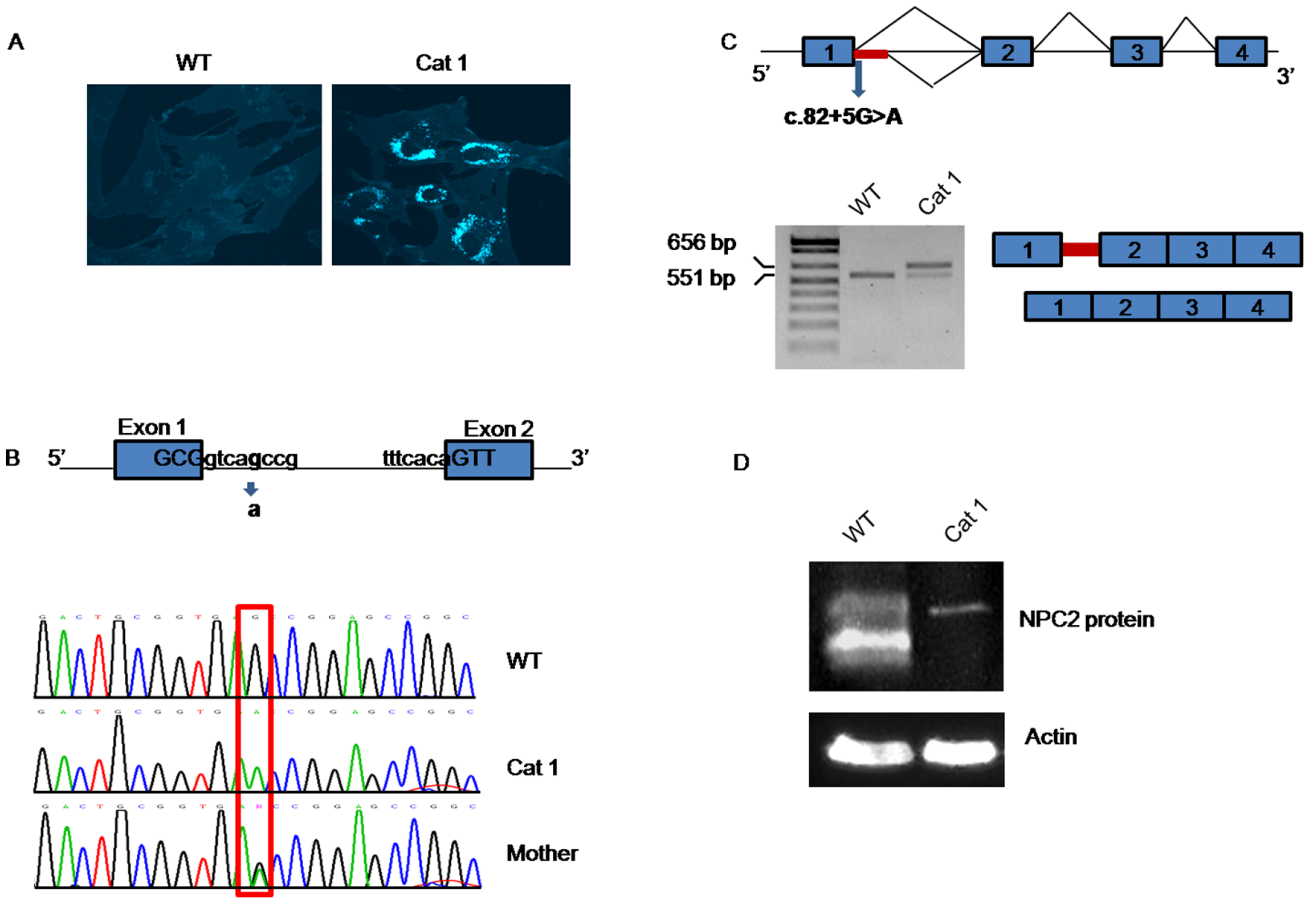
Biochemical and molecular studies. A) Filipin staining of intracellular unesterified cholesterol in cat cultured fibroblasts. B) Upper panel: schematic representation of the 5′region of *NPC2* gene. The c.82+5G>A mutation in highlighted in bold. Lowercase letters indicate intronic sequences and uppercase letters indicate exonic sequences. Lower panel: Sequencing analysis of a PCR product encompassing the exon1-intron 1 and exon 2 of *NPC2* gene showed the presence of the c.82+5G>A mutation in homozygosis in cat 1. The mother carries the same mutation in heterozygosis. C) Upper panel: schematic representation of the 5′region of *NPC2* gene. In the presence of the c.82+5G>A mutation a new 5′donor splice site located 105 bp downstream of the canonic donor splice site of intron 1 is activated. Lower panel: RT-PCR analysis of *NPC2* mRNA in cat's fibroblasts. A fragment retaining 105 bp of intron 1 is detected in fibroblasts from cat 1. D) Western blot analysis of NPC2 protein isolated from cat's fibroblasts. A protein of a higher molecular weight is detected in fibroblasts from cat 1.

Filipin staining of cultured fibroblasts from cat 1 showed massive perinuclear storage of unesterified cholesterol which is consistent with the biochemical phenotype of NPC disease (Figure 1A). Molecular analysis of *NPC1* and *NPC2* genes was then performed. No mutations were found in the *NPC1* gene, while *NPC2* analysis showed the presence of an homozygous intronic mutation located 5 nt downstream of the canonical donor splice site of exon 1 (c.82+5G>A) (Figure 1B).

The possible effect of this mutation on the mRNA splicing process was evaluated by RT-PCR and sequencing of the *NPC2* mRNA. As shown in figure 1C, the mutation affects the splicing process causing the retention of 105 bp of intron 1 in the mature mRNA, which would lead to the in frame insertion of 35 amino acids between residues 28 and 29 of the NPC2 protein (p.G28_S29ins35). In addition, a small amount of normal spliced mRNA was detected.

Western blot analysis showed the expression of an NPC2 immunoreactive protein with a higher molecular weight than the protein expressed in normal fibroblasts, consistent with the presence of a protein carrying the insertion of 35 amino acids (Figure 1D).

**Figure 2:**
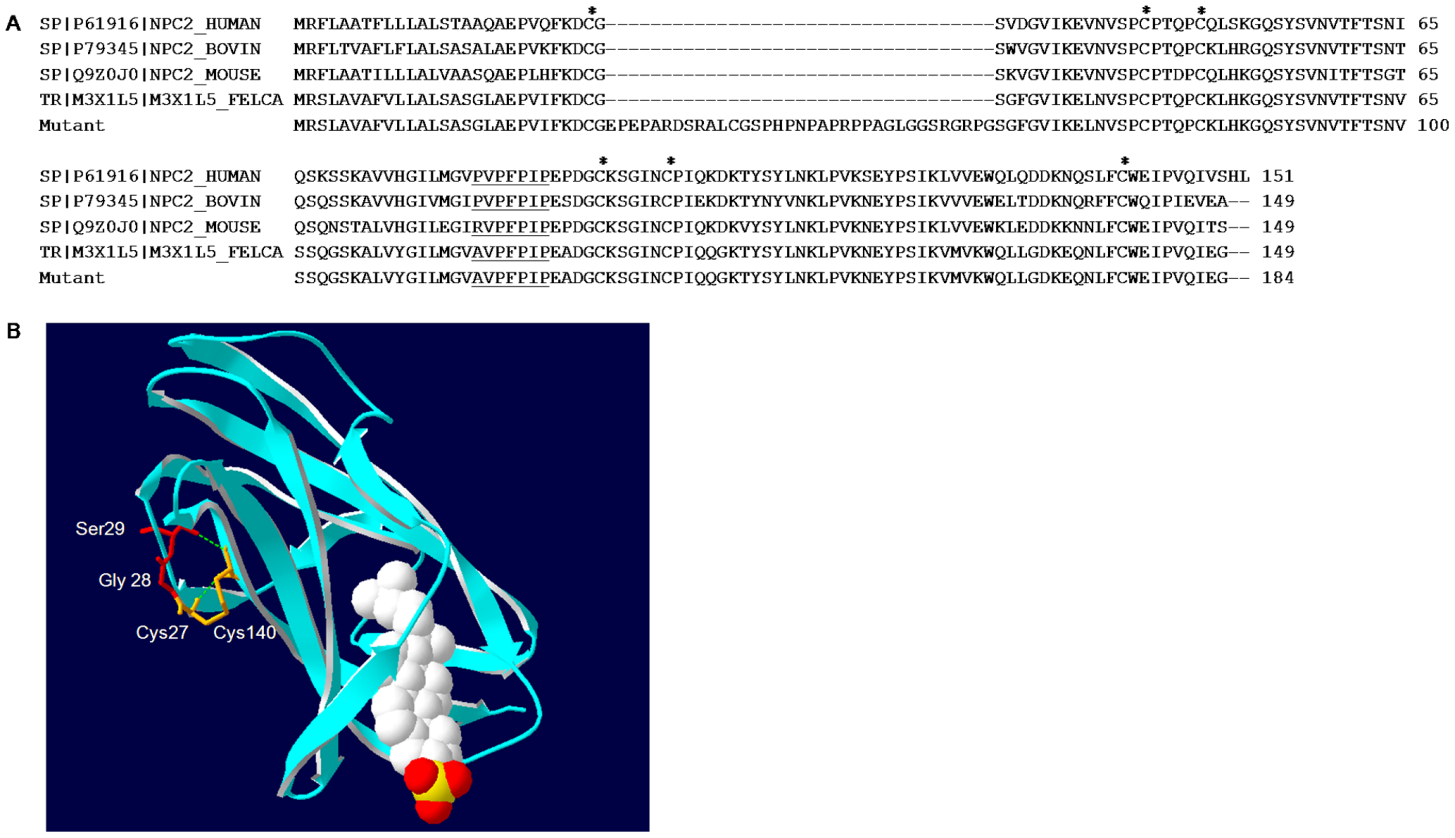
NPC2 protein alignments and structural analysis. A) Multiple NPC2 protein alignment among different mammalian orthologs The cysteine residues have been marked with asterisks while residues in the prolin-rich region have been underlined. B) Structure of bNPC2. The bNPC2 crystal structure (PDB ID 2HKA) is shown in ribbons with the amino acid residues at the boundary of the insertion site shown in red in stick representation (Gly28 and Ser29). A space-filling model of cholesterol has been bound in the proposed sterol-binding site. The cysteine residues involved in the Cys27-Cys140 disulfide bound are represneted as orange sticks. Amino acid residue numbers are calculated considering aminoacid 1, the first Metionine, as done in patient genotyping.

Multiple sequence alignment of mutant NPC2 protein with wild type feline, murine, human and bovine NPC2 showed that the primary sequence is highly conserved among many mammalian orthologs (Figure 2A). Indeed, human, bovine, murine and feline proteins have 60% identity and 27% similarity. All polypeptide sequences contain six cysteine residues, a proline-rich region (PVPFPIP) and a variable number putative Asn-linked glycosylation.

To shed further light on the possible consequences of these aminoacid changes at protein level, we performed a structural protein analysis based on the 3D model of the protein.

The 3D structure of bovine NPC2 protein and its complex with a cholesterol analog have been previously determined [20], [21]. It consists of seven β-strands arranged in two β-sheets, related by a 30° rotation. Its structure has revealed a pocket and two cavities that make a tunnel which widens to accommodate the substrate [20], [21] (Figure 2B). Three disulfide bonds connecting residues C27-C140, C42-C47 and C93-C99 stabilize the bNPC2 structure [20]. G28-S29 amino acid residues lay on protein surface. Even though the mutation does not appear to affect the cholesterol binding site, the in frame insertion might alter the conformation of the cholesterol binding pocket, since this pocket needs to expand in order to accommodate cholesterol.

In addition, DiANNA software (http://clavius.bc.edu/~clotelab/DiANNA/) was used to in silico analyze whether the mutated N-terminus would change disulfide bond patterns within NPC2. It was predicted that whereas residue 27 would bind with residue 140 in the wild type protein (score of 0.95678 on a scale of 0 to 1), residue 27 would likely bind with residue 40 in the mutated protein (score of 0.99751), which is located within the insertion. The mutation is not predicted to alter the disulfide bonds C42-C47, and C93-C99. This change may lead to protein instability. Considering this scenario it is likely that the mutated protein is partially degraded, consistent with the low levels of protein expression detected by western blot.

### Histological analysis

**Figure 3:**
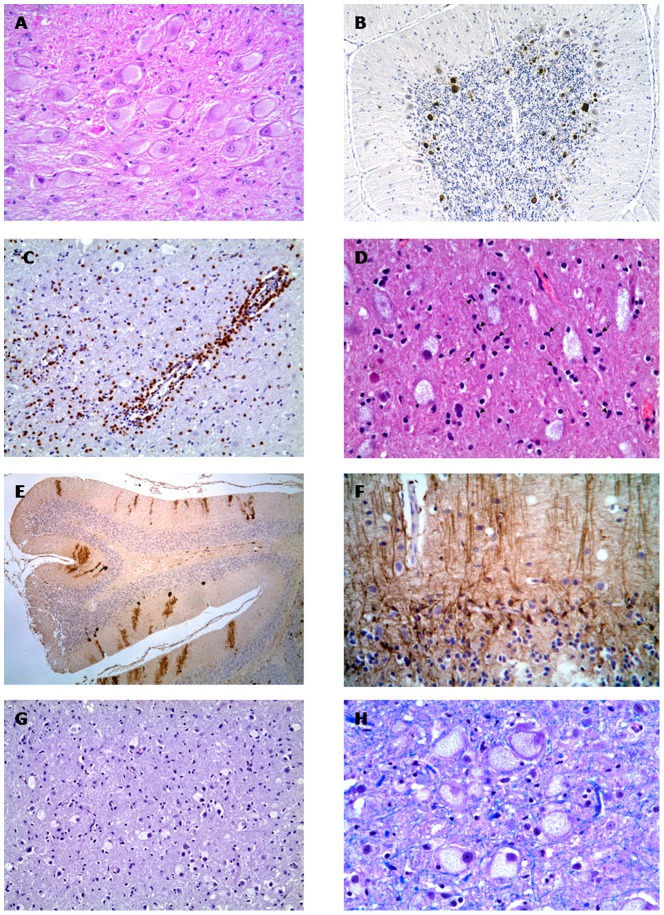
Post mortem histological analysis of central nervous system tissues. A) Brain stem: Cytoplasmic swelling of neurons with accumulation of faintly eosinophilic storage material. H&E, ×320. B) Cerebellar cortex: Spheroids within the granule cell layer are labelled with anti-ubiquitin antibody. IHC, ×125. C) Thalamus: Inflammatory infiltrate with perivascular lymphocytic cuffing. IHC for CD3, ×200. D) Thalamus: Rod-shaped activated microglial cells (arrows) are intermingled with enlarged neural cells. H&E, ×400. E) Cerebellum: Marked loss of Purkinje cells and dendrites is evidenced by calbindin immunostaining. IHC for calbindin, ×50. F) Cerebellar cortex: Loss of Purkinje cells is accompanied by gliosis in the molecular layer. IHC for GFAP, ×320. G) Thalamus: Storage material within ballooned neural cells is negative with PAS reaction. PAS, ×200. H) Brain stem: Intracytoplasmic storage material is negative with LFB staining. LFB, ×400.

Post-mortem histological examination of cat 2 revealed a diffuse storage process in neural and extraneural tissues. The brain was macroscopically unaltered and showed diffuse neuronal ballooning (Figure 3A) with Nissl substance and nuclei displacement, associated with moderate gliosis. Moderate axonal fragmentation, associated with a mild reduction of myelin staining with LFB, was observed in the subcortical and cerebellar white matter. Spheroids with accumulation of ubiquitinated aggregates were prominent in the cerebellar cortex (Figure 3B). Scattered perivascular aggregates of T lymphocytes (Figure 3C) accompanied by microglial cells proliferation were present in the thalamus and midbrain (Figure 3D). In the cerebellar cortex, Purkinje cells were markedly reduced in number (Figure 3E) and the remaining cells showed prominent intracytoplasmic storage. Proliferation of Bergmann glia expressing GFAP was also observed (Figure 3F). The vascular endothelium of the CNS showed no signs of storage. In all tissues, the storage material was faintly eosinophilic and did not stain with PAS (Figure 3G) and LFB (Figure 3H).

**Figure 4:**
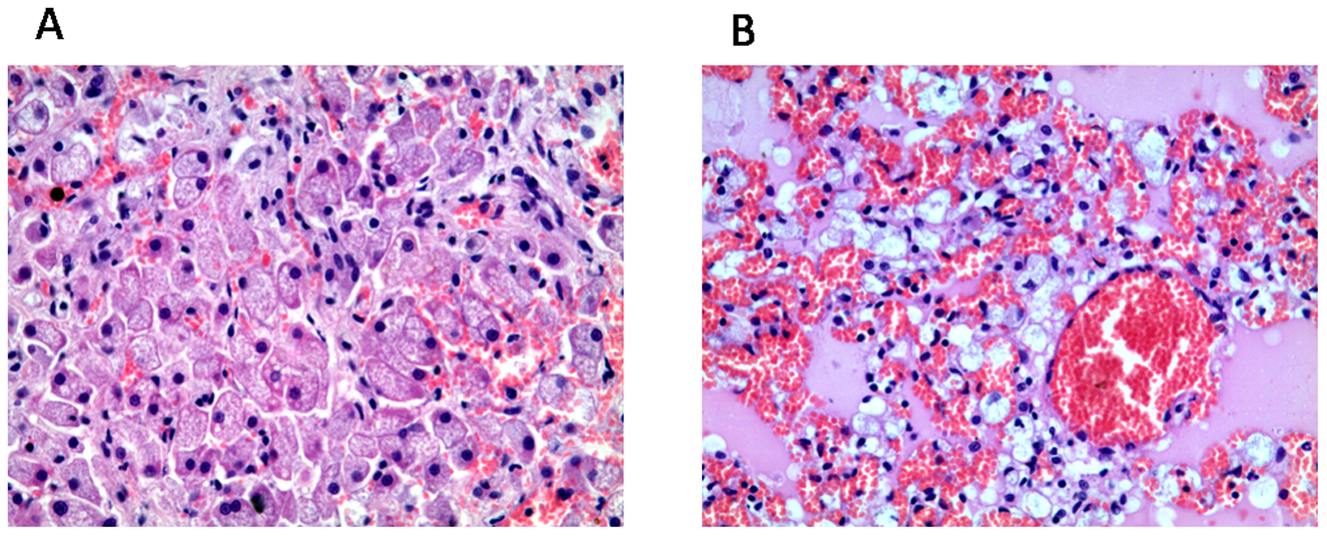
Post mortem histological analysis of liver and lung tissues. A) Liver: Hepatocytes have a foamy, vacuolated appearance due to lipidstorage. H&E, ×400. B) Lung: Accumulation of foamy macrophages accompanied by congestion and edema. H&E, ×400.

Histological examination of the liver showed the presence of swollen hepatocytes because of accumulation of small vacuoles of relatively uniform size, imparting foaminess to the cytoplasm (Figure 4A). Dispersed small foamy Kupffer cells and congestion of sinusoids were also detected. In the lungs, foamy macrophages were observed within the pulmonary interstitium and alveoli (Figure 4B). Pneumocytes and ciliated epithelial cells within airway walls appeared morphologically normal.

## Discussion

Niemann pick type C disease is a rare neurovisceral lysosomal storage disorder caused by mutations in either *NPC1* or *NPC2* genes. Most human patients present mutations in NPC1 gene, while only 5% of patients present mutations in *NPC2* gene. In humans, approximately 360 NPC1 and 22 NPC2 mutations have been described to date.

*NPC1* but not *NPC2* spontaneous mutations have been reported in different species other than humans, such us mouse, cats and dogs [11], [12], [14], [15].

The clinical features of the 2 kittens described in this report closely resemble the human NPC phenotype. The age at onset and the progression of neurological symptoms were very similar to those described in the feline model of NPC presenting mutations in *NPC1* gene [16], which resembles the juvenile form of the disease in humans [22].

Neither of the cats presented hepatosplenomegaly or pulmonary involvement. Although these two features are frequently present in NPC patients, they are absent in half of the patients reported to be affected by the juvenile/adult form of NPC2 [23], [24]. This observation further support the idea that these cats would resemble the juvenile phenotype of the human disease.

It is worth noting that the severe neonatal form of the disease is the most common one among human NPC2 patients, while *NPC2* mutations have been described only in few juvenile/adult patients [23], [24], [25], [26].

Molecular analysis of the *NPC2* gene led to the identification of a homozygous intronic mutation located 5 nt downstream of the canonical donor splice site of exon 1 (c.82+5G>A). Interestingly, a mutation affecting the same splice site (c.82+2T>C) has been described in a human patient [8]. However, although both mutations affect the *NPC2* mRNA splicing process, they lead to the transcription of different splice variants. The c.82+2T>C mutation leads to the synthesis of 3 splice variant of which one retains 57 bp on intron 1 and would generate a protein with an in-frame insertion of 19 aminoacids between 28 and 29 of the NPC2 protein (p.G28_S29ins19). Nevertheless, no NPC2 protein was detected in cells carrying this mutation.

Instead, the c.82+5G>A mutation leads to the transcription of one spliced variant retaining 105 bp of intron 1 in the mature mRNA, which would lead to the in frame insertion of 35 amino acids between residues 28 and 29 of the NPC2 protein (p.G28_S29ins35). In addition, a small amount of normal spliced mRNA was transcribed as well. However, only the aberrant *NPC2* mRNA variant carrying the 105 bp insertion seems to be translated into NPC2 immunoreactive protein. It is likely that the amount of normal *NPC2* mRNA expressed by these cells is too low to generate enough wild type protein to be detected by western blot analysis. It is worth noting that the clinical phenotype displayed by these kittens suggests the presence of some residual NPC2 function. In fact, cat 2 survived 10 months and cat 1 died at 21 months. However, whether the protein carrying an insertion of 35 aminoacids is also partially functional needs to be further investigated.
